# Arsenic trioxide inhibits transforming growth factor-β1-induced fibroblast to myofibroblast differentiation in vitro and bleomycin induced lung fibrosis in vivo

**DOI:** 10.1186/1465-9921-15-51

**Published:** 2014-04-24

**Authors:** Fayong Luo, Yan Zhuang, Mark D Sides, Cecilia G Sanchez, Bin Shan, Eric S White, Joseph A Lasky

**Affiliations:** 1 Department of Medicine, Section of Pulmonary Diseases, Critical Care and Environmental Medicine, Tulane University Health Science Center, New Orleans, LA 70112, USA; 2 Department of Internal Medicine, Division of Pulmonary and Critical Care Medicine, University of Michigan Medical School, Ann Arbor, MI, USA

**Keywords:** Arsenic trioxide, IPF, TGF-β1, Pulmonary fibrosis, PML, Bleomycin

## Abstract

**Background:**

Idiopathic pulmonary fibrosis (IPF) is a progressive disease of insidious onset, and is responsible for up to 30,000 deaths per year in the U.S. Excessive production of extracellular matrix by myofibroblasts has been shown to be an important pathological feature in IPF. TGF-β1 is expressed in fibrotic lung and promotes fibroblast to myofibroblast differentiation (FMD) as well as matrix deposition.

**Methods:**

To identify the mechanism of Arsenic trioxide’s (ATO)’s anti-fibrotic effect in vitro, normal human lung fibroblasts (NHLFs) were treated with ATO for 24 hours and were then exposed to TGF-β1 (1 ng/ml) before harvesting at multiple time points. To investigate whether ATO is able to alleviate lung fibrosis in vivo, C57BL/6 mice were administered bleomycin by oropharyngeal aspiration and ATO was injected intraperitoneally daily for 14 days. Quantitative real-time PCR, western blotting, and immunofluorescent staining were used to assess the expression of fibrotic markers such as α-smooth muscle actin (α-SMA) and α-1 type I collagen.

**Results:**

Treatment of NHLFs with ATO at very low concentrations (10-20nM) inhibits TGF-β1-induced α-smooth muscle actin (α-SMA) and α-1 type I collagen mRNA and protein expression. ATO also diminishes the TGF-β1-mediated contractile response in NHLFs. ATO’s down-regulation of profibrotic molecules is associated with inhibition of Akt, as well as Smad2/Smad3 phosphorylation. TGF-β1-induced H_2_O_2_ and NOX-4 mRNA expression are also blocked by ATO. ATO-mediated reduction in Smad3 phosphorylation correlated with a reduction of promyelocytic leukemia (PML) nuclear bodies and PML protein expression. PML-/- mouse embryonic fibroblasts (MEFs) showed decreased fibronectin and PAI-1 expression in response to TGF-β1. Daily intraperitoneal injection of ATO (1 mg/kg) in C57BL/6 mice inhibits bleomycin induced lung α-1 type I collagen mRNA and protein expression.

**Conclusions:**

In summary, these data indicate that low concentrations of ATO inhibit TGF-β1-induced fibroblast to myofibroblast differentiation and decreases bleomycin induced pulmonary fibrosis.

## Background

Idiopathic pulmonary fibrosis (IPF) is a chronic, progressive and fatal disease [1]. A recent study indicated that the 5-year survival of IPF ranges from 30% - 50%. Despite tremendous progress in our understanding of the pathogenesis of IPF, no effective medicinal treatment has been shown to improve the mortality in afflicted patients [2]. A prominent pathological feature of IPF is the formation of fibrotic foci, which consist of myofibroblasts and the extracellular matrix which they produce. Myofibroblasts are the principle effecter cells synthesizing pro-fibrotic proteins such as α-SMA, type I collagen, and fibronectin. Although multiple types of cells can differentiate into myofibroblasts, fibroblast to myofibroblast differentiation (FMD) is the major source for myofibroblast accumulation [3].

Transforming growth factor (TGF)-β1 is a potent fibrogenic cytokine and plays a crucial role in the pathogenesis of pulmonary fibrosis [4]. TGF-β1 induces FMD by activating Smad3 and Akt signaling pathways [5,6]. Over-expression of TGF-β1 using of a recombinant adenovirus vector carrying an active TGF-β1 construct is sufficient to induce pulmonary fibrosis in vivo [4]. In addition, interventions that inhibit TGF-β1 signaling have been shown to block the development pulmonary fibrosis in animal models [7]. To date there are no approved therapies to target TGF-β1 for the treatment of pulmonary fibrosis, so furthering our understanding of the profibrotic effects of TGF-β1 may lead to an effective therapy for pulmonary fibrosis.

Arsenic trioxide (ATO) has been used as a drug for more than 2000 years for the treatment of diseases including ulcers, psoriasis, and malaria [8]. In the modern era, arsenic has been shown to be effective for the treatment of various cancers, especially acute promyelocytic leukemia (APL) [9,10]. Recent studies have shown that ATO can regulate the expression of various proteins as well as pathways involved with TGF-β1 signaling. For example, ATO induces SnoN/SkiL, an inhibitory TGF-β1 regulator by affecting Smad3 nuclear transportation in ovarian carcinoma cells [11]. ATO has also been reported to induce TG-interacting factor (TGIF), which is another well-characterized Smad co-repressor for TGF-β1 responsive genes. Interestingly, ATO also degrades promyelocytic leukemia (PML) nuclear bodies and PML protein expression in various cancer cell lines [12].

PML was originally identified as a fusion partner of retinoic acid receptor alpha (RARα) in APL patients [13]. PML protein contains an N-terminus RING finger, two B-boxes and a coiled-coil domain, which are encoded by the first 3 exons of the PML gene. Seven different isoforms differ from each other in the C-terminus, which are generated by alternative splicing from exon 4 to exon 9. PML proteins exist in both the cytoplasm and nucleus. However, the majority of PML protein locates to the nucleus and forms complicated protein structures known as PML nuclear bodies. PML nuclear bodies are dynamic structures with diameters ranging from 200 nm to 1 μm. They exist in almost all mammalian cells and play important roles in DNA damage repair, transcription regulation, viral defense, control of apoptosis and senescence [14,15]. PML may also regulate TGF-β1 signaling, as cytoplasmic PML (cPML) has been shown to aid with Smad3 phosphorylation in mouse embryonic fibroblasts (MEFs) by facilitating interactions among Smad anchor for receptor activation (SARA), Smad3 and the Type-I TGF-β1 receptor [16]. However, to date the role of PML in TGF-β1-induced FMD and lung fibrosis in vivo has not been addressed.

To better understand the potential effect of ATO in regulating TGF-β1-induced FMD and lung fibrosis, we investigated how TGF-β1 signaling pathways were regulated in normal human lung fibroblasts (NHLFs) in response to treatment with ATO. We observed that ATO inhibited TGF-β1 signaling by inhibition of Smad2/Smad3 and Akt phosphorylation. We also examined the anti-fibrotic effect of ATO in vivo by using a murine bleomycin model of pulmonary fibrosis, and found that intraperitoneal administration of ATO reduced bleomycin induced pulmonary fibrosis in C57/BL6 mice.

## Methods

### Reagents and antibodies

Arsenic trioxide (Sigma-Aldrich) was prepared in 1 N NaOH at 250 mM and then diluted in sterile water for a stock concentration of 1 μM. Cell culture medium, FGM-2 and DMEM, were purchased from Lonza (Allendale, NJ) and Gibco. Human recombinant TGF-β1 was purchased from R&D systems (Minneapolis, MN). Antibodies used were: alpha-SMA (Sigma-Aldrich, 1:10,000), type-1 collagen (abcam, 1:2000), PML (Santa Cruz, 1:500), PAI-1 (peprotech, 1:2000), fibronectin (BD science, 1:500). Antibodies for Smad2, p-Samd2, Smad3, p-Smad3, Akt, p-Akt, Erk, p-Erk, p38, p-p38 were purchased from Cell signaling and used at a concentration of 1:1000.

### Cell culture

Normal human lung fibroblasts (NHLFs) were purchased from Lonza (Allendale, NJ). Cells were maintained in FGM-2 (Lonza) and only early passage cells (before passage 6) were used for all experiments. Wild-typed and PML -/- mouse embryonic fibroblasts (MEFs) were a kind gift from the laboratory of Dr. Pier Paolo Pandolfi (Beth Israel Deaconess Cancer Center) and maintained in DMEM with 20% FBS and 1% penicillin-streptomycin (Gibco). Human lung fibroblasts from control patients and IPF patients were a generous gift from the laboratory of Dr. Eric S. White (University of Michigan, Ann Arbor).

### Western blot analysis

Cells were harvested using 1x RIPA buffer (Cell signaling) with 1 mM PMSF (Sigma Aldrich). Thirty μg of protein per sample was loaded onto 4–12% Novex Tris-Glycine SDS polyacrylamide gels (Invitrogen) for electrophoresis and then transferred on polyvinylidene difluoride (PVDF) membranes (0.45 μm, Invitrogen). Membranes were then blocked in 5% Milk (BioRad) for 1 hour at room temperature and then incubated with the appropriate primary antibody overnight. Secondary antibodies and an ECL kit from (GE) were applied for generating chemiluminescent signals. All western blot data represents triplicate repeats. Densitometry analysis was performed using National Institutes of Health (NIH) ImageJ software.

### Real time quantitative PCR

Real time quantitative PCR was performed using the iCycler (Bio-Rad Laboratories, Hercules, CA), and SYBR green supermix (Bio-Rad) was employed according to the manufacturer’s instructions. mRNA expression was corrected to expression of the 36B4 housekeeping gene. Primer sequences that were employed were: h-α-SMA: Fwd: GAAGAAGAGGACAGCACT, Rev: TCCCATTCCCACCATCAC; m-α-SMA: Fwd: TGCTGACAGAGGCACCACTGAA, Rev: CAGTTGTACGTCCAGAGGCATA; h-Collagen-1: Fwd: CGGAGGAGAGTCAGGAAGG, Rev: CACAAGGAACAGAACAGAACA; m-Collagen-1: Fwd: GCCAAGAAGACATCCCTGAAG, Rev: TCATTGCATTGCACGTCATC; 36B4: Fwd: CGACCTGGAAGTCCAACTAC; Rev: ATCTGCTGCATCTGCTTG; h-CTGF: Fwd: GGCTTACCGACTGGAAGAC, Rev: AGGAGGCGTTGTCATTGG; h-PAI-1: Fwd: GGCTGGTGCTGGTGAATGC; Rev: AGTGCTGCCGTCTGATTTGTG.

### Rat tail type I collagen gel contraction assay

Rat tail type I collagen gel contraction assay was conducted as previously described [17]. Briefly, a 12-well cell culture plate was pre-coated with 5% sterile BSA for 4 hours. Rat tail type-I collagen (354236 BD biosciences) was diluted with fibroblast basal medium (FBM, Lonza, CC-3131) with 0.5% BSA into 2 mg/ml and mixed with NHLFs to reach a final concentration of 2 × 10^5^ cells/ml. One N NaOH was added as per the manufacture’s instruction. Eight hundred microliters of cell-collagen mixture were then added into each well of the culture plate and incubated at 37° for 30 minutes. Cells were incubated in FBM medium with 0.5% BSA overnight. Cells were treated with arsenic trioxide and TGF-β1 as indicated. Gel sizes were measured using the National Institutes of Health (NIH) ImageJ software. To harvest the cells from the gel, a type I collagenase (Invitrogen) was applied to dissolve the collagen gel. Briefly, trypsin was added onto the gels for 5 minutes. Type I collagenase (5 mg/ml) was then added onto the gels and they were incubated at 37° for 30 minutes. Cells were spun down and lysed using RIPA buffer for western blot analysis.

### Immnofluorescent staining

NHLFs were plated into 8 well chamber slides and treated as indicated. After washing in PBS for 5 minutes, cells were fixed by 4% paraformaldehyde (Electron Microscopy Sciences) for 10 minutes. Fixed cells were then washed in HBSS for 5 minutes 3 times before a blocking buffer (5% Goat serum + 0.5% BSA + 0.4% Triton X-100) was added for 1 hr at room temperature. Appropriate primary antibodies and secondary antibodies were then added for one hr at room temperature. A 10 minute HBSS wash times 3 was performed prior to the addition of the secondary antibody. DAPI (Invitrogen) was added for nuclear staining and prolong gold (Invitrogen) was used for preserving the signal.

For processing paraffin embedded tissue slides, slides were incubated at 60°C for 45 min. Then slides were de-paraffinized by immersion in xylene and re-hydrated. Following hydration, slides were boiled in 1x SSC for 10mins for antigen retrieval. After boiling slides were kept in hot SSC for 30 mins. at room temperature. Slides were then washed in 50 μM ammonium chloride for 10 mins., then rinsed in PBS for 10 mins. 3 times prior to application of the primary antibody.

### MTT assay

An in vitro toxicology assay kit (Sigma Aldrich) was used for this experiment. Briefly, NHLFs were plated into a 12-well plate and pretreated with ATO (10nM, 20nM) for 48 hrs. Cells were then washed with HBSS prior to addition of 1 ml of FGM. MTT (M-5655, Sigma Aldrich) was added into to media and cells were incubated at 37°C for 2 hrs. Thereafter, the culture media was removed and MTT Solubilization Solution (M-8910, Sigma Aldrich) was added. Absorbance of each well was measured at the wavelengths of 570 nm and 690 nm.

### H_2_O_2_ detection

NHLFs were treated with ATO and TGF-β1 as indicated. Then cells were incubated with 30 μM DCFH-DA at 37°C for 30 min. and washed with HBSS several times. Cells were lysed in 1 N NaOH and the intensity of fluorescence was determined using a plate reader with an excitation filter at 485 nm and an emission filter at 535 nm. The H_2_O_2_ level was calculated as the mean fluorescence intensity of each sample.

### Cytoplasmic and nuclear protein extraction

Proteins from cytoplasm and nucleus compartments were separated by using NE-PER Nuclear and Cytoplasmic Extraction Reagents (Thermo scientific, #78835). Briefly, NHLFs were harvested with trypsin-EDTA and then washed twice with PBS. Then cells were centrifuged at 500 × g for 5 minutes and supernatants were removed. Ice cold CER-I and CER-II solutions were added per the manufacturer’s instructions to separate the cytoplasmic from the nuclear compartment proteins. Western blot for GAPDH and histone 3 were used to ensure there was no contamination in each part of the extracts.

### Bleomycin and ATO treatment

All protocols for animal studies were approved by the Institutional Animal Care and Use Committee of Tulane University. C57/BL6 mice (Charles River) were separated into 5 groups with 7 mice in each group. Two days before bleomycin exposure, arsenic trioxide (1 mg/kg) was administered daily by intraperitoneal injection in the ATO-pretreatment group. PBS with the same amount of NaOH employed to solubilize the ATO was used as a control for other groups. Bleomycin (Zhejiang Hisun Pharmaceutical Co., Ltd, 2units/kg) was given by oropharyngeal aspiration as described previously (25). For the ATO-delayed treatment group, ATO was administered daily starting on day 6 following bleomycin administration. Mice were sacrificed on day 14 after administration of bleomycin. Left lungs were fixed for trichrome staining and right lungs were harvested and homogenized in liquid nitrogen for real time quantitative PCR and western blot analysis.

### Total collagen quantification

Left lungs were paraffin-embedded and tissue slides were prepared for trichrome staining. An Aperio slide scanner (Aperio, CA) was used to scan the tissue slides per the manufacturer’s instruction. The whole lung tissue section on a single slide was scanned and collagen content was calculated using an internal PPC Collagen (2) RWB program. A ratio of total positive value to the total number value was used to represent collagen expression.

### Statistic anaylysis

Statistic analysis was conducted using ANOVA followed by the Bonferroni *post hoc* test. Data are presented as the mean (±SEM) and represent multiple experiments performed in triplicate.

## Results

### ATO inhibits TGF-β1 induced fibrotic markers

**Figure 1:**
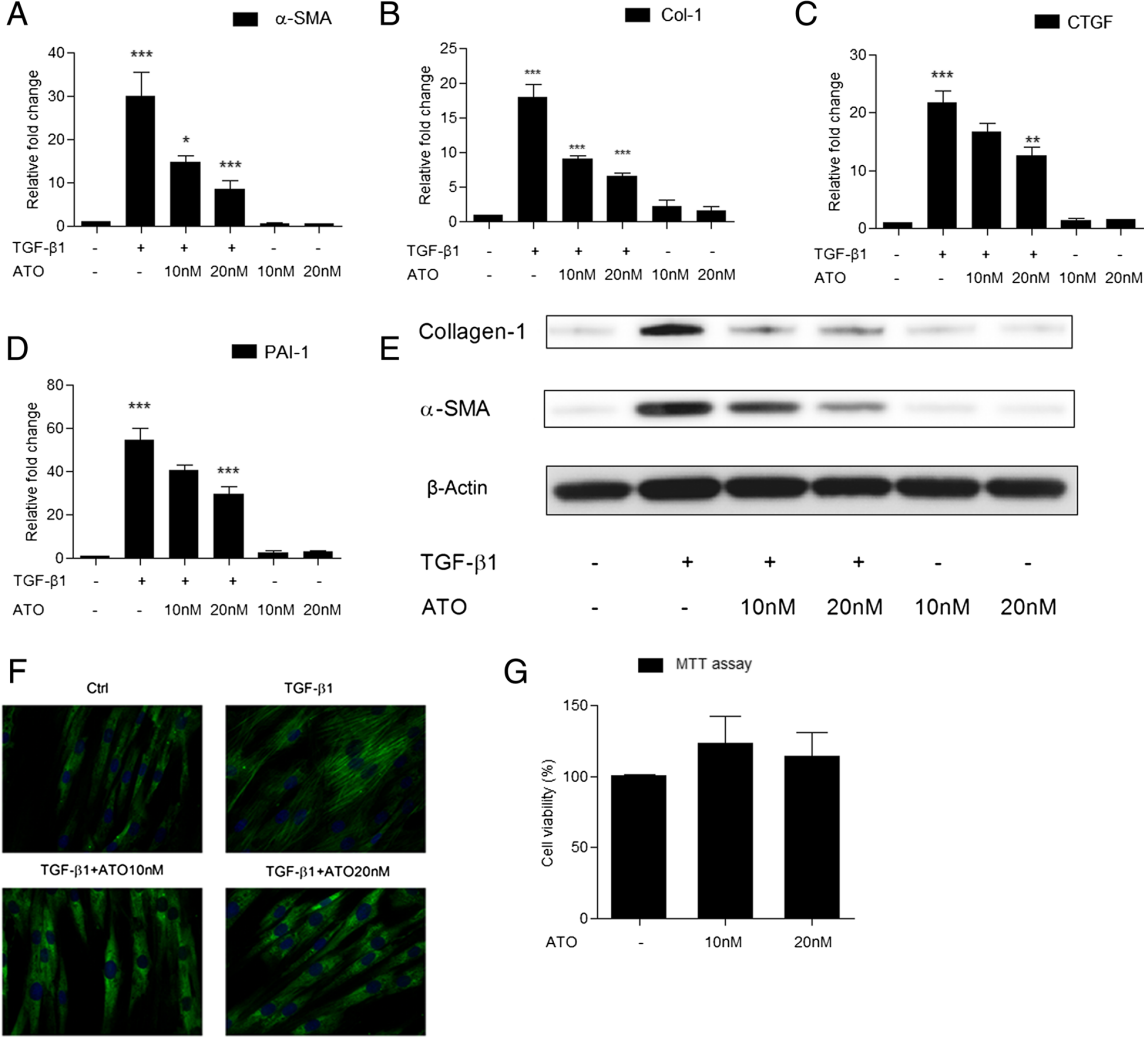
**ATO inhibits TGF-β1-induced α-SMA and collagen expression. (A-D)** Normal human lung fibroblasts (NHLFs) were serum starved overnight and treated with Arsenic trioxide (ATO) for 24 hrs, then treated with TGF-β1 (1 ng/ml) for 24 hrs. α-SMA, Collagen-1, PAI-1 and CTGF mRNA expression levels were evaluated by TGF-β1 and ATO inhibited TGF-β1’s effect. **(E)** TGF-β1-induced α-SMA and collagen-1 protein expression was blocked by ATO in NHLFs. **(F)** Immunofluorescent staining showed TGF-β1-induced α-SMA fiber formation was reduced by ATO. **(G)** An MTT assay showing that ATO does not affect NHLF viability. RT-PCR Data represent results from three independent experiments with duplicate repeats. Western blot and immunofluoresent staining data represent consistent trend in three independent repeats with the best image quality. *P value < 0.05, **P value <0.01, ***P value < 0.001.

Myofibroblasts play a crucial role in formation of fibroblastic foci [3]. TGF-β1 induces FMD and increases α-SMA and type I collagen expression. To examine whether ATO inhibits TGF-β1-induced FMD, NHLFs were pretreated with very low concentrations of ATO (10nM or 20nM) for 24 hrs and then exposed to TGF-β1 (1 ng/ml) for another 24 hrs. α-SMA and type I collagen mRNA expression were induced by TGF-β1 (α-SMA: 29.90 ± 5.45; Col-1: 18.02 ± 1.85; p < 0.05), however these effects were diminished by ATO treatment (α-SMA: 14.76 ± 1.38 & 8.47 ± 2.06 vs. 29.90 ± 5.45; p < 0.05; Col-1: 9.10 ± 0.40 & 6.52 ± 0.52 vs. 18.02 ± 1.85; p <0.05) (Figure 1A, 1B). Connective tissue growth factor (CTGF) and plasminogen activation inhibitor 1 (PAI-1) were also induced by TGF-β1 (CTGF: 21.73 ± 1.54; PAI-1: 54.40 ± 5.48; p < 0.05) and are thought to play important roles in pulmonary fibrosis [6,18]. Pretreatment of ATO decreased TGF-β1-induced expression of CTGF and PAI-1 mRNA (CTGF: 12.47 ± 1.60 vs. 21.73 ± 1.54; p < 0.05; PAI-1: 29.19 ± 3.95 vs. 54.40 ± 5.48; p < 0.05) (Figure 1C, 1D). Western blots were performed on proteins derived from duplicate wells. α-SMA and type I collagen protein expression were induced by TGF-β1, and pretreatment with ATO diminished this effect (Figure 1E). Furthermore, ATO (10nM, 20nM) blocked TGF-β1 induced α-SMA and type I collagen protein expression in fibroblasts extracted from control and IPF patient lungs (Additional file 1: Figure S1A, S1B). A delayed ATO treatment experiment in which ATO (10nM, 20nM) was given to NHLFs after 24 hrs of TGF-β1 (1 ng/ml) exposure also blocked TGF-β1 induced α-SMA and type I collagen protein expression (Additional file 1: Figure S1C).

NHLFs have a spindle-shaped morphology while myofibroblasts are more stellar shaped cells and express α-SMA in fibrils [19]. NHLFs were pretreated with ATO (10nM, 20nM) for 24 hrs and then treated with TGF-β1 (1 ng/ml) for another 24 hrs, to test whether ATO affects TGF-β1-induced. α-SMA fiber formation. Immunofluorescent staining demonstrated that ATO pretreatment decreased TGF-β1 induced incorporation of α-SMA into fibrils (Figure 1F). ATO is a well-characterized inducer of apoptosis in APL as well as non-malignant cell lines [20,21]. Recent studies have shown that ATO induces pulmonary fibroblast growth inhibition at a concentration of 50 μM [22]. However the concentration of ATO used in our studies are several orders of magnitude lower than the concentration employed in the manuscripts mentioned above. To examine whether 10nM or 20nM concentrations of ATO inhibit NHLF cell viability, cells were treated with ATO for 48 hrs and protein was harvested for western blot. An MTT assay revealed no significant differences in cell viability when cells were exposed to these low concentrations of ATO (Figure 1G).

### ATO inhibits TGF-β-induced fibroblast contractile activity

**Figure 2:**
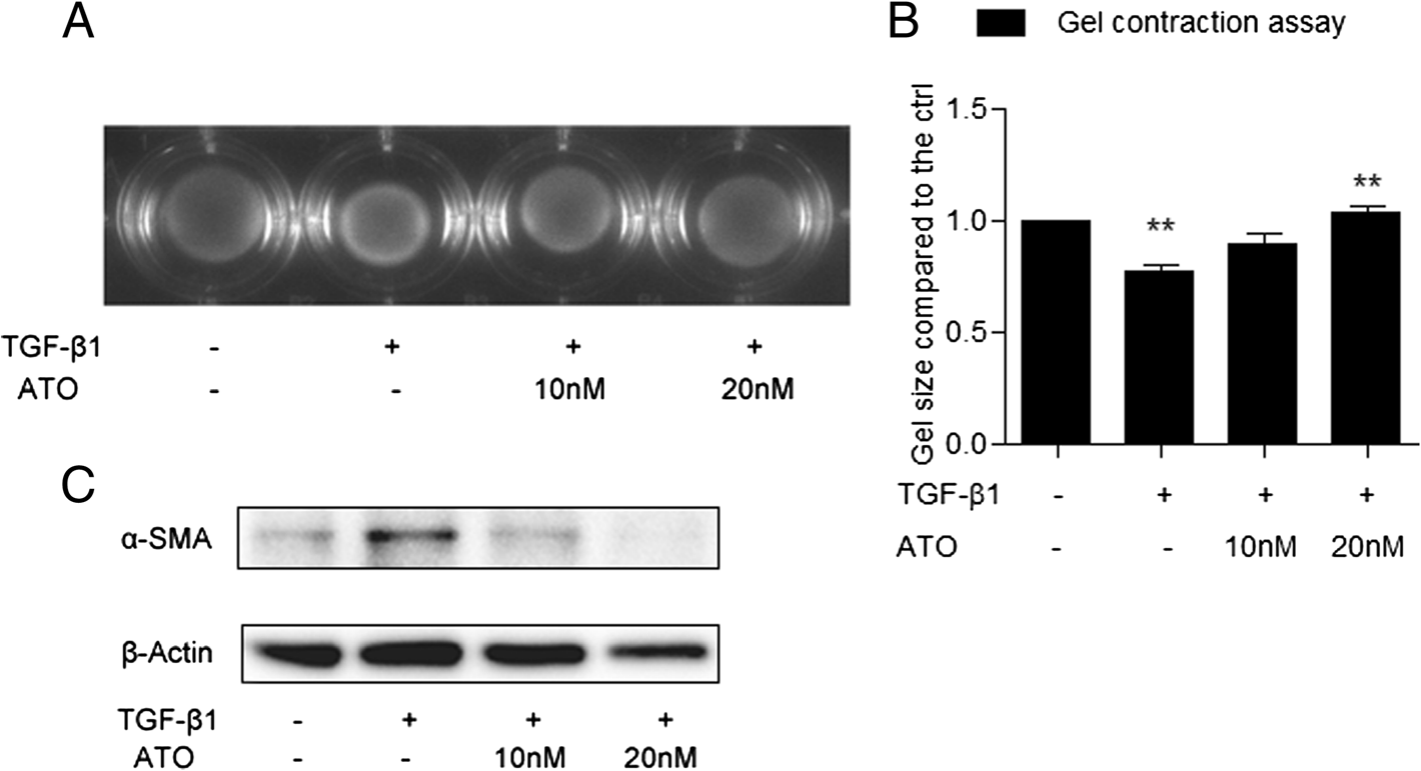
**ATO inhibits TGF-β-induced fibroblast contractile activity in rat tail type-I collagen gel. (A)** NHLFs were cultured in rat tail type I collagen gels (2 mg/ml) and pre-treated with ATO for 24 hrs. Cells were exposed to TGF-β1 (1 ng/ml) for another 48 hrs. TGF-β1treatment decreased the size of the gel while ATO pre-treatment blocked the reduction in gel size. **(B)** A summation of the percentage of gel surface area in each well of Figure 2A to the control gel surface area. **(C)** NHLFs were cultured in rat tail type I collagen gels and treated as described above. Gels were digested using type-I collagenase (5 mg/ml) and cells were harvested in SDS loading buffer for western blot. α-SMA protein expression was induced by TGF-β1 and ATO abrogated the induction. Data represent results from three independent experiments with triplicate repeats. *P value < 0.05, **P value <0.01, ***P value < 0.001.

Myofibroblasts have greater contractile activity compared to fibroblasts owing to the elevated expression level of α-SMA [23]. A type I collagen gel contraction assay was conducted to assess whether ATO regulates TGF-β1-induced contraction in NHLFs, NHLFs were cultured in type-I collagen gel as described in Methods. Cells were pretreated with ATO (10nM or 20nM) for 24 hrs and then treated with TGF-β1 (1 ng/ml) for another 48 hrs. Treatment with TGF-β1 resulted in a decrease in the size of the collagen gel (78% ± 2%; p < 0.05), indicative of an increase in contractility, whereas pretreatment of ATO blocked this effect (89% ± 5% & 104% ± 4% vs. 78% ± 2%; p < 0.05) (Figure 2A, 2B). We also harvested the NHLFs from collagen gels to correlate the contractile activity with α-SMA expression. Western blot demonstrated that TGF-β1 increases α-SMA protein expression, and that pretreatment of ATO inhibits this effect (Figure 2C).

### ATO inhibits Smad2/Smad3 and Akt phosphorylation

**Figure 3:**
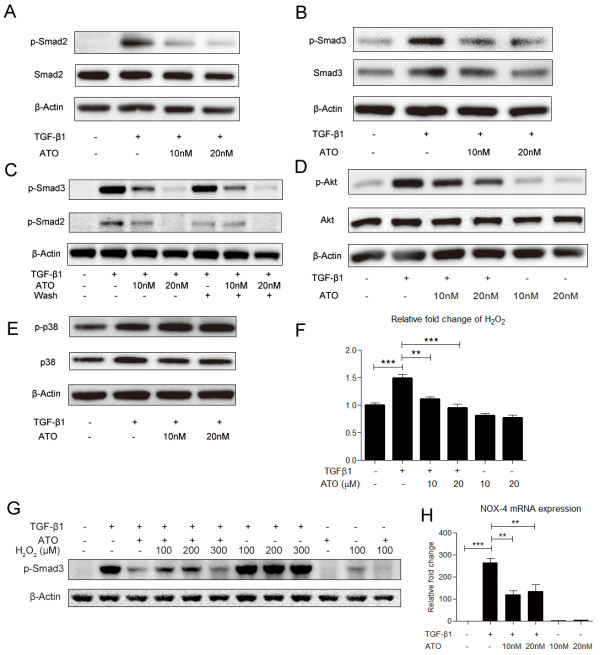
**ATO inhibits Smad2/Smad3 and Akt phosphorylation but does not affect p38 phosphorylation. (A-B)** NHLFs were pre-treated with ATO for 24 hrs. Then cells were treated with TGF-β1 (1 ng/ml) for 30 mins. TGF-β1 did not affect total Smad2 and Smad3 expression, but did increase their phosphorylation. TGF-β1 induced Smad2 and Smad3 phosphorylation were blocked by ATO. **(C)** NHLFs were pre-treated with ATO for 24 hrs, and then washed 5 times with PBS to remove ATO from the media. Thereafter cells were exposed to TGF-β1 (1 ng/ml) for 30 mins. The results indicate that the effects of ATO on TGF-β1 induced Smad2 and Smad3 phosphorylation are not due to disruption of the of the TGF-β1 ligand. **(D)** NHLFs were pre-treated with ATO for 24 hrs., and then treated with TGF-β1 (1 ng/ml) for 12 hrs. Akt phosphorylation was induced by TGF-β1 and this induction was diminished by ATO. **(E)** NHLFs were pre-treated with ATO for 24 hrs, then treated with TGF-β1 (1 ng/ml) for 30 mins. ATO increased TGF-β1 induced p38 phosphorylation. **(F)** TGF-β1 induces H_2_O_2_ in NHLFs. ATO at the indicated concentrations did not induce H_2_O_2_, but did diminish TGF-β1-induced H_2_O_2_ production. **(G)** NHLFs were pre-treated with ATO for 24 hrs, then treated with TGF-β1 (1 ng/ml) with or without H_2_O_2_ for 30 mins. Smad3 phosphorylation was up-regulated by H_2_O_2._**(H)** TGF-β1 induced NOX-4 mRNA expression in NHLFs, whereas ATO at the indicated concentrations inhibited TGF-β1-induced NOX-4 mRNA expression. Western blot data represents consistent trend in three independent repeats with the best image quality. RT-PCR data represent results from three independent experiments with duplicate repeats. *P value < 0.05, **P value <0.01, ***P value < 0.001.

TGF-β1 induces FMD by activating Smad2/3 and Akt phosphorylation [5,6]. To investigate TGF-β1 signaling pathways that may be affected by ATO, we pretreated NHLFs with ATO (10nM or 20nM) for 24 hrs and then treated the cells with TGF-β1 for 30 mins. TGF-β1 induced Smad2/Smad3 phosphorylation, however, the level of phosphorylation was decreased in the ATO pretreatment group (Figure 3A, 3B). To investigate whether the effect of ATO on Smad phosphorylation was on account of ATO inactivating TGF-β1 in cell culture medium, NHLFs were pretreated with ATO (10nM or 20nM) for 24 hrs, then the cells were washed 5 times with PBS to remove ATO from the media prior to the addition of TGF-β1. Smad2/Smad3 phosphorylation was inhibited when ATO was removed prior to treatment with TGF-β1 (Figure 3C). This indicates that the inhibitory effects of ATO on TGF-β1 Smad phosphorylation were not due to chemical inactivation of the TGF-β1 ligand. Next we investigated the effects of ATO on TGF-β1-induced Akt phosphorylation. NHLFs were pretreated with ATO for 24 hrs and then exposed to TGF-β1 for another 12 hrs. Pretreatment of ATO decreased TGF-β1-mediated Akt phosphorylation (Figure 3D).

To assess whether ATO down-regulates TGF-β1-driven phosphorylation on a global level, we also evaluated P38 phosphorylation, which is also involved in TGF-β1 signaling [24]. NHLFs were pretreated with ATO (10nM or 20nM) for 24 hrs and treated with TGF-β1 for 30 mins. p38 phosphorylation was induced by TGF-β1 and ATO pretreatment did not diminish its phosphorylation (Figure 3E). This indicates that ATO does not globally affect phosphorylation. Erk phosphorylation was also assessed, however, we did not observe an increase in Erk phosphorylation in the NHLFs in response to TGF-β1 over several time points, nor a diminution in the baseline Erk phosphorylation in response to ATO.

### ATO blocks TGF-β1 induced H_2_O_2_ and NOX-4 mRNA expression

Reactive oxygen species (ROS), especially H_2_O_2_ plays an important role in the derivation of TGF-β1-mediated fibrotic phenotypes [18]. To investigate whether low doses of ATO regulate H_2_O_2_ levels in NHLFs, we pretreated the cells with ATO (10nM or 20nM) for 24 hrs and then exposed them to TGF-β1 for another 12 hrs. These low concentrations of ATO did not induce H_2_O_2_ (0.81 ± 0.04 & 0.77 ± 0.04; p > 0.05), and to the contrary, TGF-β1-induced H_2_O_2_ expression was blocked by ATO (1.11 ± 0.04 & 0.95 ± 0.07 vs. 1.49 ± 0.06; p < 0.05) (Figure 3F). To investigate whether ATO down-regulated TGF-β1 induced Smad3 phosphorylation by reducing H_2_O_2_ expression, NHLFs were pretreated with ATO (20nM) for 24 hrs and then exposed to TGF-β1 with or without H_2_O_2_ (100 μM, 200 μM, and 300 μM) for an addional 30 mins. H_2_O_2_ (100 μM) partially restored Smad3 phosphorylation reduced by ATO (Figure 3G). The NADPH oxidase (NOX) proteins generate H_2_O_2_ by transferring electrons to oxygen, and NOX-4 has been reported to mediate myofibroblast differentiation [25,26]. To determine how ATO regulates TGF-β1 induced NOX-4 expression, NHLFs were pretreated with ATO (10nM or 20nM) for 24 hrs and then treated with TGF-β1 (1 ng/ml) for another 24 hrs. TGF-β1 induced a marked up-regulation of NOX-4 mRNA (264.80 ± 19.29; p < 0.05), however pre-treatment with ATO significantly blunted this effect (119.50 ± 17.66 & 136.20 ± 28.32 vs. 264.80 ± 19.29; p < 0.05) (Figure 3H).

### ATO causes a reduction in PML protein and PML nuclear bodies in NHLFs

**Figure 4:**
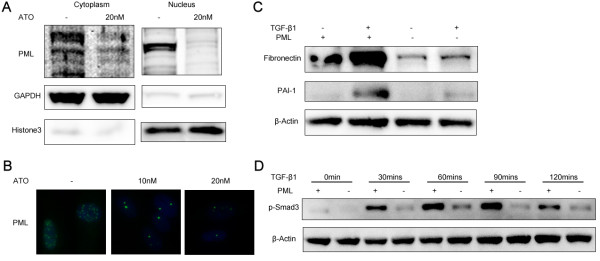
**TGF-β1 induced PAI-1, fibronectin and Smad3 phosphorylation are impaired in PML -/- MEFs. (A)** NHLFs were treated with ATO (20nM) for 24 hrs. Cytoplasmic and nuclear compartments were separated and extracted. PML protein expression was decreased in both the cytoplasm and nucleus in response to ATO treatment. **(B)** Immunofluorescent staining shows that PML bodies in NHLFs were decreased in response to ATO treatment. **(C)** Wild type and PML-/- mouse embryonic fibroblasts (MEFs) were treated with TGF-β1 (1 ng/ml) for 24 hrs. Fibronectin-EDA and PAI-1 protein expression was induced by TGF-β1 in WT-MEFs but not PML -/- MEFs. **(D)** Wild-type and PML -/- MEFs were treated with TGF-β1 (1 ng/ml) and cells were harvested at the indicated time points. Smad3 phosphorylation was induced in WT-MEFs and the level of phosphorylation was markedly decreased in PML -/- MEFs. Western blot data represents consistent trend in three independent repeats with the best image quality.

ATO has been reported to degrade PML proteins and PML nuclear bodies in various cancer cell lines [12], and cytoplasmic PML (cPML) has been shown to play an essential role in Smad-dependent TGF-β1 signaling [16]. To investigate whether ATO decreases PML protein expression in NHLFs, NHLFs were treated with ATO (20nM) for 24 hrs. Both cytoplasmic and nuclear PML were down-regulated by ATO (Figure 4A). PML nuclear bodies are dynamic structures in the nucleus that are responsible for transcription regulation, DNA damage repair, and viral defense. PML is the backbone of PML bodies and interacts with other sumoylated proteins to form PML nuclear bodies. NHLFs were treated with ATO for 24 hrs to investigate whether ATO reduces PML nuclear bodies in NHLFs, and immunofluorescent staining showed that PML body intensity was dramatically decreased in response to ATO treatment (Figure 4B).

### PML is essential for TGF-β signaling in MEFs

To test whether PML is essential for TGF-β1 signaling and Smad phosphorylation, wild type MEFs and PML -/- MEFs were treated with TGF-β1 (1 ng/ml) for 24 hrs and harvested for western blot analysis. TGF-β1 induced PAI-1 and fibronectin protein expression in wild-type MEFs; however the induction decreased in PML -/- MEFs (Figure 4C). TGF-β1 induces the phosphorylation of both Smad2 and Smad3 but the profibrotic effect induced by TGF-β1 is largely dependent on Smad3 activation [27]. To investigate whether PML plays a role in Smad3 activation, wild type MEFs and PML -/- MEFs were treated with TGF-β1 (1 ng/ml) for the indicated time points. Smad3 phosphorylation was induced by TGF-β1 in the wild type MEFs and this event was markedly decreased in PML -/- MEFs (Figure 4D).

### ATO inhibits bleomycin induced lung fibrosis in C57BL/6 mice

**Figure 5:**
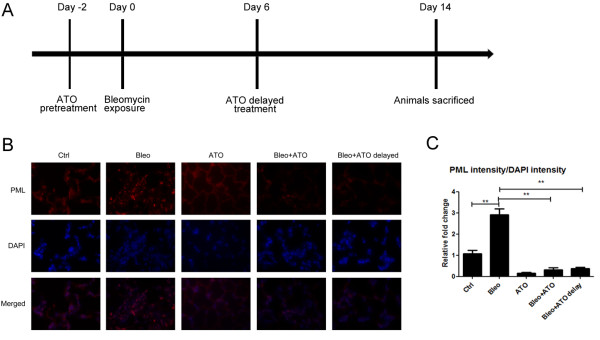
**Daily injection of ATO reduces PML body in C57BL/6 mouse lungs. (A)** Bleomycin (2units/kg) and ATO (1 mg/kg) were administered to C57BL/6 mice (n = 7) as indicated. Bleomycin was administered by oropharyngeal aspiration. ATO was administered daily by intra-peritoneal injection. **(B)** Mice were sacrificed 14 days after bleomycin administration. Bleomycin induced PML body formation, while ATO disrupted their formation, in mouse lungs. **(C)** Densitometric quantification of PML body intensity for Figure 5B. *P value < 0.05, **P value <0.01, ***P value < 0.001.

The bleomycin model is a well-established model of pulmonary fibrosis and is TGF-β1 dependent [28]. To examine whether ATO inhibits bleomycin-induced fibrosis in vivo, ATO (1 mg/kg) and control diluent (PBS + NaOH) were delivered to mice (5 groups, n = 7) by daily intraperitoneal injection. In the pre-treatment group ATO was started 2 days before bleomycin administration, and in the delayed treatment group ATO was started 6 days after the bleomycin administration. Bleomycin (2units/kg) and dilutent control (PBS) were given by oropharyngeal administration. Mice were sacrificed 14 days after treatment with of bleomycin (Figure 5A). Immunofluorescent staining showed that bleomycin increased PML body intensity while ATO decreased this effect in vivo (0.32 ± 0.08 & 0.37 ± 0.04 vs. 2.83 ± 0.16; p < 0.05) (Figure 5B, 5C).

**Figure 6:**
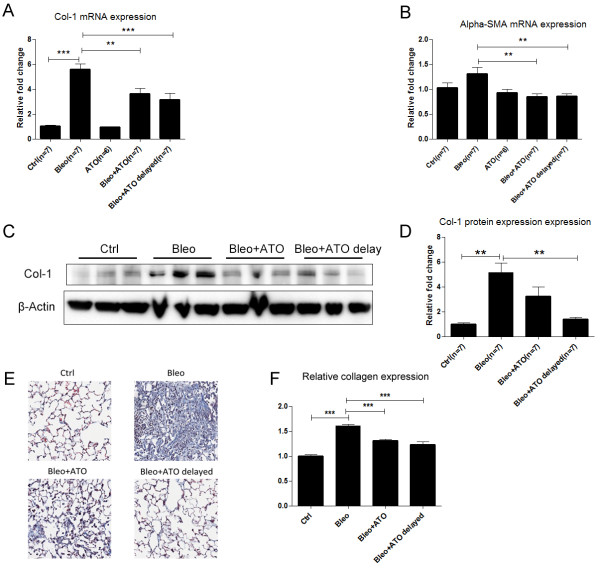
**ATO inhibits bleomycin induced lung fibrosis phenotypes in C57BL/6 mice. (A, B)** C57BL/6 mice were exposed to bleomycin and ATO as described in Figure 5. Type-1 collagen and α-SMA mRNA expression were induced by bleomycin and this effect was diminished by ATO. **(C, D)** Increased Type-I collagen protein expression in response to bleomycin treatment was blocked by ATO, and was statistically significant. **(E)** Representative histology showing that bleomycin induced inflammation and collagen deposition in mouse lungs, and these features were reduced by ATO treatment. **(F)** Total collagen expression on lung sections was evaluated using the Aperio software scanning system. Bleomycin induced total collagen expression and ATO treatment decreased this effect.

To evaluate the effects of ATO on level of fibrosis induced by bleomycin, murine left lungs were homogenized in liquid nitrogen and mRNA was harvested for RT-qPCR. Type-I collagen mRNA expression was induced by bleomycin whereas both pretreatment and delayed ATO treatment reduced this induction (3.61 ± 0.47 & 3.15 ± 0.51 vs. 5.60 ± 0.42; p < 0.05) (Figure 6A). α-SMA mRNA expression was not up-regulated by bleomycin to a statistically significant level (1.31 ± 0.12; p > 0.05). However the expression level of α-SMA was significantly decreased both groups treated with ATO (0.84 ± 0.07 & 0.85 ± 0.05 vs. 1.31 ± 0.12; p < 0.05) (Figure 6B). Total protein was also harvested to evaluate lung type-I collagen protein expression. Bleomycin increased type-I collagen protein expression, while the pretreatment and delayed treatment of ATO decreased its expression (Figure 6C-D). To assess lung histological changes induced by bleomycin, Mason’s trichrome staining was applied to lung sections. Bleomycin induced robust collagen deposition in lungs. In contrast, pretreatment and delayed treatment with ATO appeared to preserve lung architecture (Figure 6E, Additional file 2: Figure S2). Tissue slides were scanned using an Aperio slide scanner to quantify total collagen expression. Bleomycin treatment up-regulated total collagen expression while pretreatment and delayed treatment with ATO diminished this effect (1.31 ± 0.02 & 1.23 ± 0.06 vs. 1.61 ± 0.03; p < 0.05) (Figure 6F).

**Figure 7:**
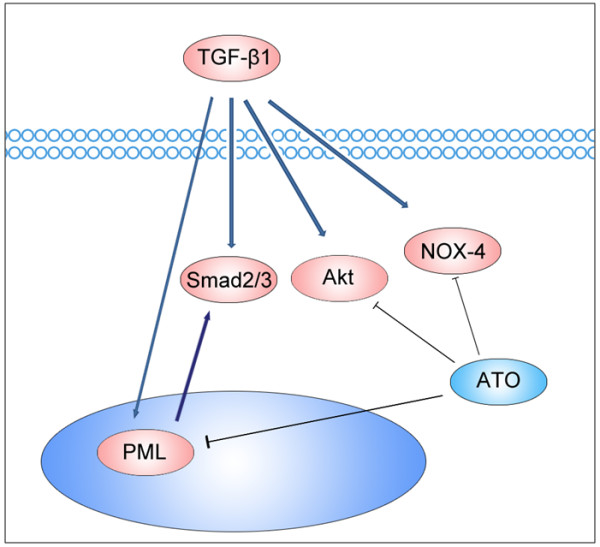
**Depiction of in vitro experiments summarizing how ATO regulates TGF-β1 signaling.** ATO blocks TGF-β1 induced fibroblast to myofibroblast differentiation by suppressing Smad2, Smad3 and Akt phosphorylation. ATO also blocks TGF-β1 induced NOX-4 mRNA expression and therefore abrogates TGF-β1-induced H_2_O_2_. Furthermore, ATO degrades PML bodies and suppresses PML protein expression, which regulates Smad3 phosphorylation and may play an important role in the pathogenesis of pulmonary fibrosis.

In summary, ATO inhibits TGF-β1 induced fibroblast to myofibroblast differentiation by blocking multiple signaling pathways including Smad2, Smad3 and Akt phosphorylation, PML expression, NOX-4 mRNA and H_2_O_2_ generation in vitro (Figure 7). Moreover, low dose ATO blocks bleomycin-induced lung fibrosis, and to date there is evidence that this correlates with a reduction in PML bodies.

## Discussion

In this study, we have demonstrated that ATO inhibits TGF-β1 induced FMD, as well as type-I collagen and α-SMA expression. We also have shown that ATO reduces bleomycin-mediated pulmonary fibrosis in mice. As for the mechanism, we found that ATO down-regulates Smad2/Smad3 and Akt phosphorylation in vitro. We also showed that ATO decreased PML expression in NHLFs, which could in turn regulate Smad3 phosphorylation. ATO has very recently been shown to be protective against bleomycin- induced fibrosis by subcutaneous injection in BALB/c mice [29], which further supports our hypothesis that ATO could be used as an anti-fibrotic agent. Our study provides more detailed and profound description of ATO’s anti-fibrotic effect by illustrating the mechanisms in vitro, and employs a 5-times lower dose (1 mg/kg) of ATO, as well as, a more bleomycin more susceptible mouse strain for the in vivo studies [30]. What is more, our experimental design included early and late administration of ATO following lung injury, and makes an association with PML body expression.

The dose of ATO is critical for its potential therapeutic application for the treatment of pulmonary fibrosis. At concentrations in the micromolar range, ATO induces ROS and promotes apoptosis for fibroblasts and other primary cells [22,31-33]. ATO can also induce acute airway epithelial injury by altering ATP-dependent Ca^2^+ signaling [34]. Furthermore, intravenous injection of ATO (1 mg/kg) was reported to induce cardiac fibrosis [35]. However, ATO concentration in the nanomolar range, as we used in this study, is less toxic. The MTT assay indicates that ATO at 10nM and 20nM does not change the NHLF viability. The report of cardiac fibrosis had not been published at the time we conducted our in vivo experiments, and although we did not note a cardiac effect on chest dissection we were not specifically looking for one. ROS, especially H_2_O_2_, are considered to be profibrogenic and are induced by TGF-β1 through a mechanism that involves up-regulation of NOX-4 activity [26]. However, ATO at 10nM and 20nM did not induce H_2_O_2_, but conversely blocked TGF-β1 induced NOX-4 mRNA and H_2_O_2_ expression. In addition, in the ATO treatment alone group, there were no observed untoward histological changes. Of relevance, the concentration we used in our study is several orders of magnitude lower than the clinical application of ATO for the treatment of APL [36].

Fibroblast to myofibroblast differentiation (FMD) is a crucial step for the genesis of myofibroblasts. Myofibroblasts are the effecter cells for producing extra-cellular matrix in fibroblastic foci which lead to loss of alveolar function of IPF lungs [37]. Myofibroblasts secrets α-SMA, a stress fiber not only affects the compliance of lungs but also works as a signal transduction molecule to regulate extracellular matrix proteins production [3]. Myofibroblasts also contribute to abnormal epithelial functions and induce epithelial apoptosis by secreting pro-inflammatory cytokines [38]. Instead of directly inducing apoptosis of fibroblasts, we have shown that low dose of ATO blocked expression of multiple TGF-β1-induced myofibroblast markers as well as mRNA levels of potent profibrotic cytokines and proteins such as CTGF and PAI-1.

Smad and Akt activation are two key pathways involved in the development of TGF-β1-induced fibrogenic phenotypes. A previous study has shown that ATO at concentrations of 10 μM diminishes Smad3 protein expression [11]. Although knocking down basal Smad3 expression might be beneficial for blocking TGF-β1 induced FMD, loss of Smad3 has been reported to correlate with enlargement of airspaces and development of emphysema [27]. In this study, we show that ATO in the nanomolar range inhibits TGF-β1 induced Smad3 phosphorylation, but does not decrease Smad3 expression. ATO has also been reported to inhibit Akt phosphorylation at a concentration of 3 μM in lymphoma B cells [21]. Consistent with that study, we observed that a low concentration of ATO inhibits TGF-β1 induced Akt phosphorylation in NHLFs. The experiment in which ATO was washed away prior to adding TGF-β1 excludes the possibility of TGF-β1 ligand inactivation as an explanation for the inhibition of Smad and Akt phorphorylation. Moreover, ATO does not inhibit phosphorylation in a global level, as the experimental data show that Erk and p38 phosphorylation are not diminished in response to ATO.

To investigate how Smad3 phosphorylation is affected by ATO, we focused on PML, a protein reported to be degraded by ATO [12]. Consistent with a previous study [16], we found that TGF-β1-induced PAI-1 and fibronectin expression as well as Smad3 phosphorylation were all impaired in PML -/- MEFs. To further test our hypothesis that PML knockdown by ATO may be responsible for an impaired TGF-β1 signaling in NHLFs, a “rescue” experiment to transfect PML plasmid into NHLFs in an attempt to restore TGF-β1 signaling was considered. However, ATO can efficiently induce the oligomerization of PML, which promotes its ubiquitination and degradation [39]. Thus, we did not pursue this approach because exogenous PML would be expected to be degraded by ATO in a similar manner. In addition to the important role of cPML in TGF-β1 signaling, PML may have profound impact on the pathogenesis of IPF via induction of cellular senescence through interactions with p53 and Rb [40]. Accelerated epithelial senescence has been observed in IPF lungs compared to control lungs [40]. Furthermore, PML functions as a negative regulator of hTERT and therefore contributes to short telomere length [41], and short telomeres have been reported to be a risk factor for IPF [33,42]. Taken together, PML may play a key role in the pathogenesis of IPF, but further experiments will need to be conducted to test this concept. In addition to PML, we have also shown that H_2_O_2_ might also play a role in ATO’s reduction on TGF-β1 induced Smad3 phosphorylation. H_2_O_2_ (100 μM) partially restored ATO reduced Smad3 phosphorylation. How ATO regulates H_2_O_2_ and Nox4 expression is an area of interest to us for future studies.

Lastly we have shown that ATO inhibits bleomycin-induced fibrosis in vivo. We have shown that 1 mg/kg of ATO is able to significantly reduce the expression of PML bodies in C57BL/6 mouse lungs, as it blocks bleomycin induced type-1 collagen, and diminishes the basal level of α-SMA expression in mouse lungs. The delayed treatment group has a slightly better effect compared with the pre-treatment group. One possible explanation for this may be associated with the massive DNA damage induced by bleomycin to the epithelial cells [28] and PML bodies are actively involved in the DNA repair process. Thus, the absence of PML bodies at the time of DNA injury could lead to sustained epithelial cell dysfunction and possibly a higher level of pro-inflammatory cytokines [14]. Future experiments, are planned that will employ PML KO mice in murine models of pulmonary fibrosis.

## Conclusions

In summary, this work demonstrates that ATO effectively inhibits TGF-β1 induced FMD in vitro and reduces lung fibrogenesis in vivo. Although the ATO concentrations employed in these experiments were low and ATO is already an FDA approved drug, it is unclear whether or not trials using low dose ATO for the treatment of recalcitrant and deadly diseases such as IPF would be considered an acceptable form of therapy.

## Competing interests

The authors declare that they have no competing interests.

## Authors’ contributions

FL - Study design, qRT-PCR, western blot, immunofluorescent staining, gel contraction assay, bleomycin administration and ATO injection, data analysis and draft of the manuscript. YZ - bleomycin administration and animal care. MDS - Study design, preparation of ATO and draft of the manuscript. CS - Study design. BS - Study design. ESW - Study design and provide IPF fibroblasts and protocols for cell culture. JAL - Study design and draft of the manuscript. All authors read and approved the final manuscript.

## Supplementary Material

Additional file 1: Figure S1 ATO inhibits TGF-β1 induced fibrotic proteins expression in control and IPF lung fibroblasts as well as diminishing established fibrotic responses in NHLFs. **(A)** Lung fibroblasts extracted from 2 control patients were serum starved overnight and treated with ATO (10nM, 20nM) for 24 hrs, then treated with TGF-β1 (1 ng/ml) for 24 hrs. α-SMA, and Collagen-1 were induced by TGF-β1, and ATO inhibited TGF-β1’s effect. **(B)** ATO blocked TGF-β1 (1 ng/ml) induced α-SMA and Collagen-1expression in lung fibroblasts derived from 2 patients with IPF. Data for both part A and B are representative of consistent effects in both cell lines. **(C)** NHLFs were treated with TGF-β1 (1 ng/ml) for 24 hrs., then exposed to ATO (10nM, 20nM) for another 24 hrs. ATO blocked TGF-β1 induced α-SMA and Collagen-1 expression. Click here for file

Additional file 2: Figure S2 Representative histology of mouse lungs in response to bleomycin and ATO treatment. Bleomycin (2units/kg) and ATO (1 mg/kg) were administered to C57BL/6 mice (n = 7) as described in the methods section. Mice were sacrificed 14 days after bleomycin administration. Representative histology (trichrome staining) illustrating that bleomycin induced lung inflammation and fibrosis was reduced by ATO treatment. Click here for file
