# Supplementary figures and images for: Arsenic trioxide inhibits transforming growth factor-β1-induced fibroblast to myofibroblast differentiation in vitro and bleomycin induced lung fibrosis in vivo
Source: Respir Res. 2014 Apr 24;15(1):51. doi: 10.1186/1465-9921-15-51 (PMC4113202; doi:10.1186/1465-9921-15-51)

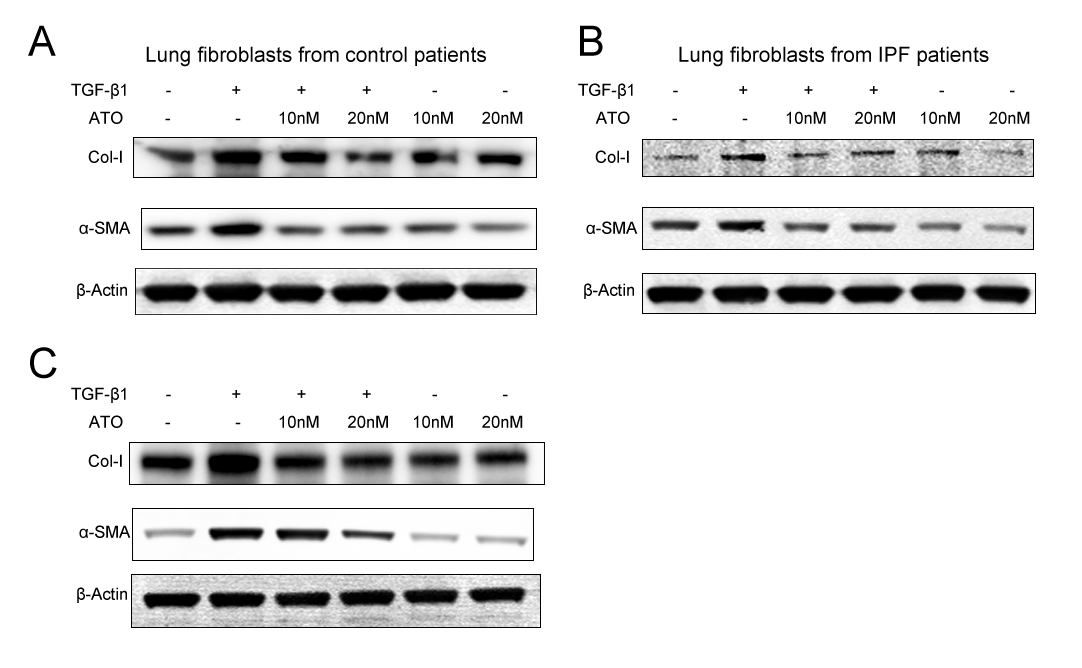

Supplement: Additional file 1: Figure S1 — ATO inhibits TGF-β1 induced fibrotic proteins expression in control and IPF lung fibroblasts as well as diminishing established fibrotic responses in NHLFs. (A) Lung fibroblasts extracted from 2 control patients were serum starved overnight and treated with ATO (10nM, 20nM) for 24 hrs, then treated with TGF-β1 (1 ng/ml) for 24 hrs. α-SMA, and Collagen-1 were induced by TGF-β1, and ATO inhibited TGF-β1’s effect. (B) ATO blocked TGF-β1 (1 ng/ml) induced α-SMA and Collagen-1expression in lung fibroblasts derived from 2 patients with IPF. Data for both part A and B are representative of consistent effects in both cell lines. (C) NHLFs were treated with TGF-β1 (1 ng/ml) for 24 hrs., then exposed to ATO (10nM, 20nM) for another 24 hrs. ATO blocked TGF-β1 induced α-SMA and Collagen-1 expression. [file 1465-9921-15-51-S1.tiff]

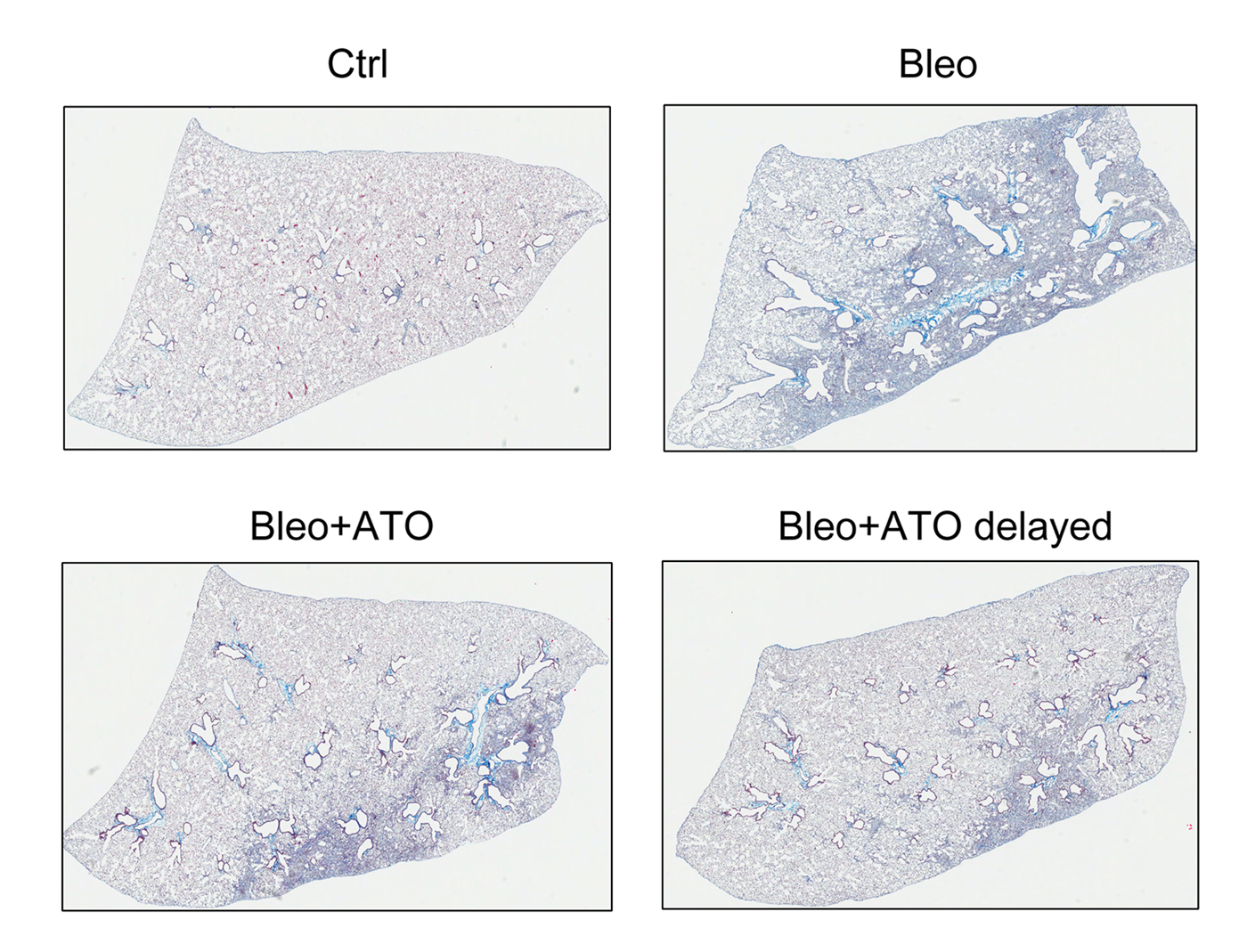

Supplement: Additional file 2: Figure S2 — Representative histology of mouse lungs in response to bleomycin and ATO treatment. Bleomycin (2units/kg) and ATO (1 mg/kg) were administered to C57BL/6 mice (n = 7) as described in the methods section. Mice were sacrificed 14 days after bleomycin administration. Representative histology (trichrome staining) illustrating that bleomycin induced lung inflammation and fibrosis was reduced by ATO treatment. [file 1465-9921-15-51-S2.tiff]
